# The Effect of Hypothermia Therapy on Cortical Laminar Disruption following Ischemic Injury in Neonatal Mice

**DOI:** 10.1371/journal.pone.0068877

**Published:** 2013-07-23

**Authors:** Hiroyuki Kida, Sadahiro Nomura, Mizuya Shinoyama, Makoto Ideguchi, Yuji Owada, Michiyasu Suzuki

**Affiliations:** 1 Department of Systems Neuroscience, Graduate School of Medicine Yamaguchi University, Ube, Japan; 2 Department of Neurosurgery, Graduate School of Medicine Yamaguchi University, Ube, Japan; 3 Department of Organ Anatomy, Graduate School of Medicine Yamaguchi University, Ube, Japan; University of South Florida, United States of America

## Abstract

Hypothermia has been proposed as a treatment for reducing neuronal damage in the brain induced by hypoxic ischemia. In the developing brain, hypoxic ischemia-induced injury may give rise to cerebral palsy (CP). However, it is unknown whether hypothermia might affect the development of CP. The purpose of this study was to investigate whether hypothermia would have a protective effect on the brains of immature, 3-day old (P3) mice after a challenge of cerebral ischemia. Cerebral ischemia was induced in P3 mice with a right common carotid artery ligation followed by hypoxia (6% O_2_, 37°C) for 30 min. Immediately after hypoxic ischemia, mice were exposed to hypothermia (32°C) or normothermia (37°C) for 24 h. At 4 weeks of age, mouse motor development was tested in a behavioral test. Mice were sacrificed at P4, P7, and 5 weeks to examine brain morphology. The laminar structure of the cortex was examined with immunohistochemistry (Cux1/Ctip2); the number of neurons was counted; and the expression of myelin basic protein (MBP) was determined. The hypothermia treatment was associated with improved neurological outcomes in the behavioral test. In the normothermia group, histological analyses indicated reduced numbers of neurons, reduced cortical laminar thickness in the deep, ischemic cortical layers, and significant reduction in MBP expression in the ischemic cortex compared to the contralateral cortex. In the hypothermia group, no reductions were noted in deep cortical layer thickness and in MBP expression in the ischemic cortex compared to the contralateral cortex. At 24 h after the hypothermia treatment prevented the neuronal cell death that had predominantly occurred in the ischemic cortical deep layers with normothermia treatment. Our findings may provide a preclinical basis for testing hypothermal therapies in patients with CP induced by hypoxic ischemia in the preterm period.

## Introduction

Cerebral palsy (CP) leads to developmental disabilities, cognitive dysfunction, and sensorimotor impairment [1], [2]. Previous studies have suggested that preterm infants born at a gestational age between 23–32 weeks have a higher risk for CP than infants carried to full-term [3]. One of the most common causes of CP is brain injury induced by hypoxic ischemia (HI). Brain development occurs slightly later in rodents than in humans. Thus, rodent models of neonatal HI have been widely used to study the pathophysiology of CP in premature birth [4]–[6].

It has been reported that, in neonatal mice, HI could induce neuronal excitotoxicity, oxidative stress, and inflammation. These states led to neuronal death, via necrosis, apoptosis [7], [8], and reduced myelination [9]. This resulted in reduced brain volume in the ischemic hemisphere compared to the contralateral hemisphere [10], [11]. However, histological details of the changes in cortical structure have not been elucidated.

Clinically, therapeutic hypothermia has been introduced for treating full-term and near-term infants with HI [12]–[14]. Previous reports have demonstrated that hypothermia reduced death rates and severe disability among survivors, but other endpoints have remained elusive. Busto *et al.* first reported the effects of hypothermia therapy in animal experiments [15]. Later, many studies reported that post-ischemic hypothermia could protect neonatal rodents from HI-induced brain injury [16]–[18]. Other studies have shown in animal models that applying hypothermia during the acute phase reduced inflammation and edema, which subsequently led to a reduction in neuronal loss in the hippocampus by preventing apoptosis [19], [20]. Recently, we found that brain cooling attenuated abnormal cerebral hyperactivities (epileptic discharges) induced by photothrombotic ischemia and drug infusion [21], [22]. However, we lack an understanding of the effect of hypothermia therapy on long-term morphological alterations in the cortex and neurofunctional outcomes in mice after neonatal ischemic injury. In this study, we used a postnatal, 3-day old (P3), HI mouse model to investigate how hypothermia therapy might ameliorate hypoxic brain injury during the neonatal period.

## Materials and Methods

ICR mice (Chiyoda kaihatsu, Japan) were housed in individual plastic cages (40 × 25 × 25 cm) and maintained at a constant temperature (22 °C) under a 12-h light/dark cycle with water and food provided *ad libitum*. Investigators responsible for surgical procedures or treatments were blinded to the mouse group assignments. All experiments were performed according to the Guidelines for Animal Experimentation of Yamaguchi University School of Medicine, and approved by the Institutional Animal Care and Use Committee of Yamaguchi University.

### Ischemia Model

At P2–P3, ICR mice of both genders (n = 67) underwent ligation of the right common carotid artery, as described previously [23]. Detection of reduced cerebral blood flow (CBF) in the ipsilateral hemisphere with laser speckle imaging (OMEGA ZONE, OZ-1) confirmed the carotid artery ligation. The pups were allowed to recover in an incubator for 1 h. The pups were then placed in acrylic chambers with a hypoxic atmosphere of 6% O_2_/94% N_2_ for 30 min and the containers were submerged in a 37°C water bath to maintain normal body temperature, as demonstrated in a previous study [11]. During this procedure, the mortality was 13.4% (9 of 67). After hypoxic exposure, the pups were randomly divided into two groups of normothermia and hypothermia. Both groups were placed in chambers that were submerged in water baths; the baths were maintained at a stable water temperature of either 37°C (normothermia) or 32°C (hypothermia). Mice remained in the submerged chambers for 24 h without the dam, as described previously [17]. The brain temperature was monitored by inserting a thermocouple into the cerebral cortex at the end of the 24-h normothermia/hypothermia treatment. Subsequently, the pups were returned to their dams.

### Behavioral Test

#### Rotarod test

To test how HI treatment in the neonatal period affected development, we administered the rotarod test (ENV577, Med Associates Inc.) to mice at 4 weeks of age. The test was conducted daily for 4 consecutive days, and mice were allowed 2 attempts (5 min each) for each test. The rotarod was set to an acceleration mode, where it increased from 4 to 40 rpm over 5 min. The average duration of rod-riding was recorded each day.

#### Beam balance test

The beam was 1750 mm long and 19 mm wide and was placed 700 mm above the floor. The beam was alternately placed 13 mm to the left or right of the wall (mice are generally more inclined to traverse the beam when a wall is present next to the beam). Performance was graded from 1 to 6, where 1 = the mouse fell off the beam; 2 = the mouse was unable to traverse the beam, but remained sitting across the beam; 3 = the mouse traversed the beam, but the affected hindlimb did not contribute to forward locomotion; 4 = the mouse traversed the beam with more than 50% foot slips; 5 = the mouse traversed the entire beam with only a few foot slips; and 6 = the mouse traversed the entire beam with no foot slips.

### Histology

The animals were euthanized with an overdose of anesthesia. Mice were then perfused transcardially with phosphate-buffered saline (PBS), followed by 4% paraformaldehyde in phosphate buffer (PB). The brain was removed and post-fixed in 4% paraformaldehyde/30% sucrose in PB for 4–12 h. For frozen sectioning, the brain was embedded in OTC compound. Then, 40 µm-thick, frozen coronal sections were cut on a cryostat and immersed in PBS. Each section included the cortex and striatum (1.10 mm to 0.14 mm from the bregma). For paraffin sectioning, the brain was embedded in paraffin, and then cut into 4-µm coronal sections with a microtome. Sections were affixed to silane-coated glass slides.

#### Immunohistochemistry

In preparation for immunohistochemistry, sections were washed in PBS for 5 min three times. To visualize neurons, sections were incubated with blocking solution provided in the Vector® M.O.M.™ Immunodetection Kit (Vector Laboratories Inc., Burlingame, CA), then we added the mouse anti-NeuN antibody (MAB377; Millipore 1∶100). To visualize the myelinated fibers, sections were incubated with 5% normal goat serum for 20 min, then we added the rat anti-MBP (ab7349; Abcam 1∶100). To detect NeuN-positive cells and MBP-positive areas, we then applied avidin-biotin horseradish peroxidase (ABC kit, Vector Laboratories), followed by diaminobenzidine tetrahydrochloride (DAB) To visualize the laminar structure, coronal sections were stained with rat anti-Ctip2 antibody (ab18465; Abcam; 1∶200), rabbit anti-CDP (Cux1) antibody (sc-13024; Santa Cruz Biotechnology; 1∶200). This was followed by incubation with Alexa Flour 488 conjugated anti-rabbit antibody and Alexa Flour 594 conjugated anti-rat antibody (Molecular Probes Inc.), respectively. The histological images were acquired on a Keyence BZ-9000 fluorescence microscope (Keyence, Osaka). The dynamic cell count program (Keyence, Osaka) was used to count the number of NeuN-positive cells and measure the laminar area. To evaluate the degree of demyelination, images were digitized with NIH imaging software (Image J).

#### TUNEL staining

To detect apoptosis with TUNEL staining, brain sections were prepared from mice on postnatal day 4. Sections were deparaffinized and heated in citrate buffer (pH 6.0) for antigen retrieval. ApopTag fluorescein was used with the In Situ Apoptosis Detection Kit (Millipore; S7110). Briefly, sections were incubated in equilibration buffer for 10 min, and terminal deoxynucleotidyl transferase (TdT) enzyme and dUTP-digoxigenin were then added to the sections and incubated in a moist chamber at 37°C for 60 min. The reaction was stopped, and the sections were washed in PBS. Sections were then incubated with anti-digoxigenin-peroxidase solution.

### Data Analysis

To evaluate the number of neurons, the total numbers of NeuN-positive cortical neurons in the cortex and striatum were counted. For this analysis, the cortical area was defined as the region surrounded by a vertical line to the cortical surface between the cingulum and the rhinal fissure. MBP staining was evaluated, as described previously [9], in terms of the ratio of optical density values measured in the right and left (R:L) hemispheres. In brief, for each brain sample, the number of pixels of MBP stain in the right hemisphere was compared to the number of pixels in the left hemisphere to calculate the R:L ratio. The superficial (upper) and deep cortical layers were defined as the regions visualized with the Cux1 and Ctip2 stains, respectively.

### Statistical Analysis

All results are expressed as means±standard error of the mean. Differences between groups were compared with the Mann-Whitney test or Wilcoxon test, where appropriate. *P*-values <0.05 were considered statistically significant.

## Results

**Figure 1:**
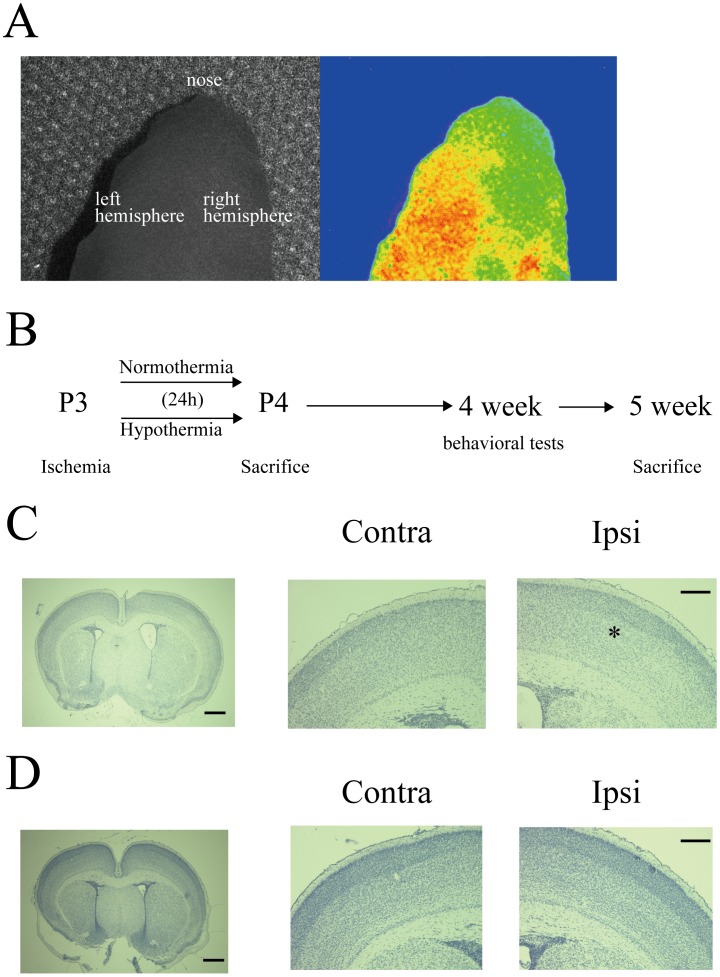
Examination of hypoxic ischemia injury in predevelopmental mouse brain. *A*: Cerebral blood flow immediately after carotid artery ligation in a P2–P3 mouse. *B*: Experimental paradigm. Hypoxic injury was followed by 24 h of normothermia or hypothermia, and mice were sacrificed at the indicated time points (P4, 5 weeks). Note that behavioral tests were conducted at 4 weeks of age. *C, D*: Representative examples of hematoxylin staining for normothermia (C) or hypothermia (D) treated mice. In the normothermia group, the boundary was obscure between the superficial layers and the deep layers (white asterisk). Scale, 500 µm. High magnification images of the contralateral (*left*) and ipsilateral (*right*) hemisphere. Scale, 200 µm.

Laser speckle imaging, performed after the right common carotid artery ligation, showed an obvious decrement in CBF in the ischemic hemisphere (Fig. 1A). After 24 h of normothermia/hypothermia (Fig. 1B), the mean brain temperatures were 31.5±0.17°C in the hypothermia group (n = 10) vs. 35.7±0.15°C for the normothermia group (n = 9) (Mann-Whitney test, *P*<0.01). To identify the extent of brain injury caused by HI and 24 h normothermia/hypothermia treatments, brain samples were stained with hematoxylin. In the normothermia group, the boundary was obscure between the superficial and deep layers in the ischemic cortex, but cortical organization was normal in the contralateral cortex (Fig. 1C). In contrast, in the hypothermia group, normal laminar structures were observed in the both ischemic and contralateral cortices (Fig. 1D).

**Figure 2:**
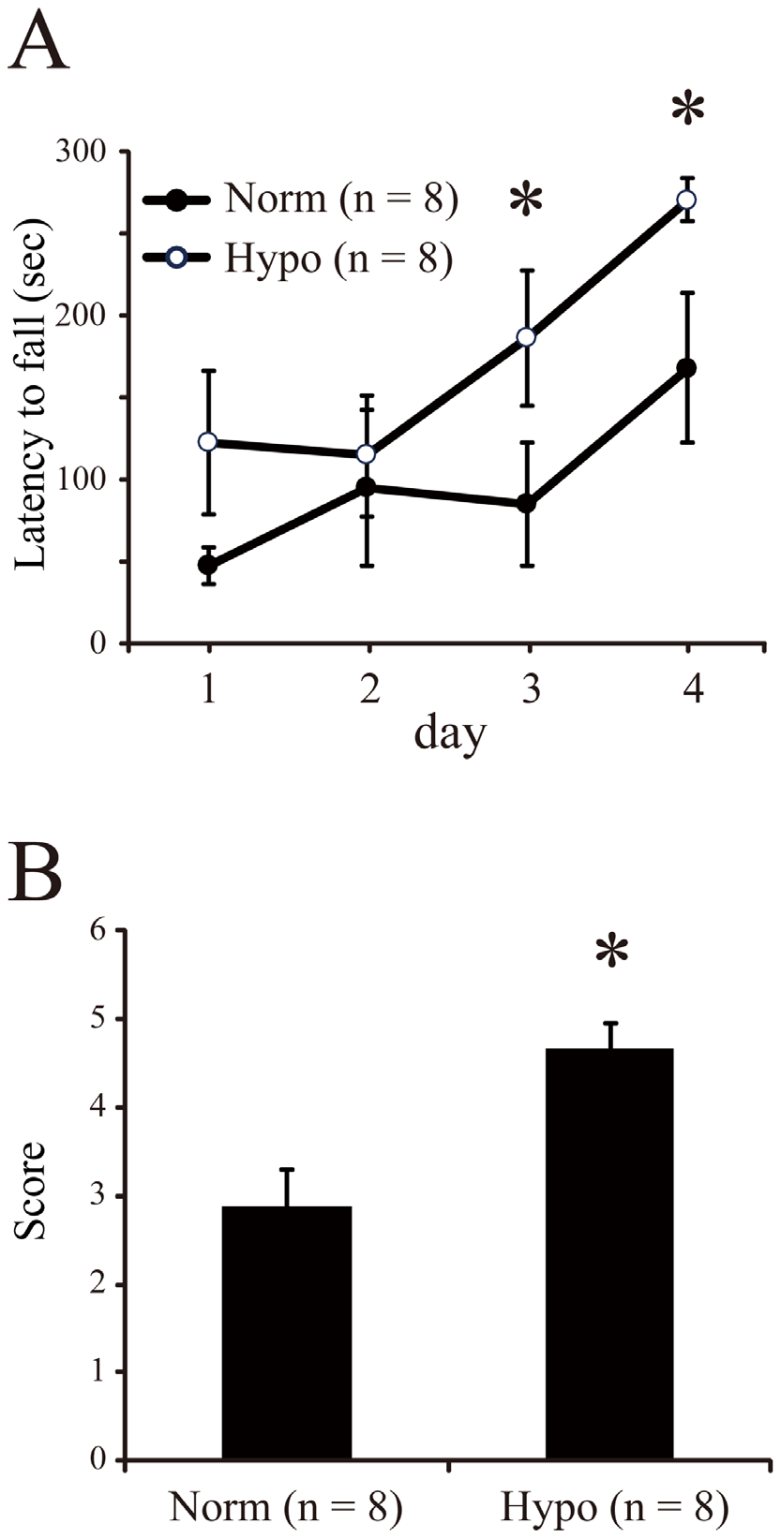
Behavioral tests for assessing brain damage in mice subjected to early hypoxic ischemia injury. *A*: Latency (mean±SE) to falling from the accelerating rotarod barrel. Normothermia (black, n = 8) and hypothermia (white, n = 8) groups were tested on 4 consecutive days. *B*: Scores in the beam balance test for normothermia (n = 8) and hypothermia groups (n = 8). * *P<*0.05 (Mann-Whitney test).

To assess motor function, mice were tested with rotarod and beam balance tests at 4 weeks of age. Rotarod performance was evaluated on four consecutive days. The average latency to falling from the rotating barrel is shown in Figure 2A. Longer latencies indicated better motor performance. Both groups showed improved performance over the four days, but the hypothermia group showed significantly longer latencies than the normothermia group after 3 days (Mann-Whitney test, *P*<0.05). The beam balance test was performed to assess motor and coordination performances. The test scores were 2.88±0.41 and 4.63±0.32 for the normothermia and hypothermia groups, respectively (Mann-Whitney test, *P*<0.05, Fig. 2B). This also indicated that hypothermia led to better motor control than normothermia.

**Figure 3:**
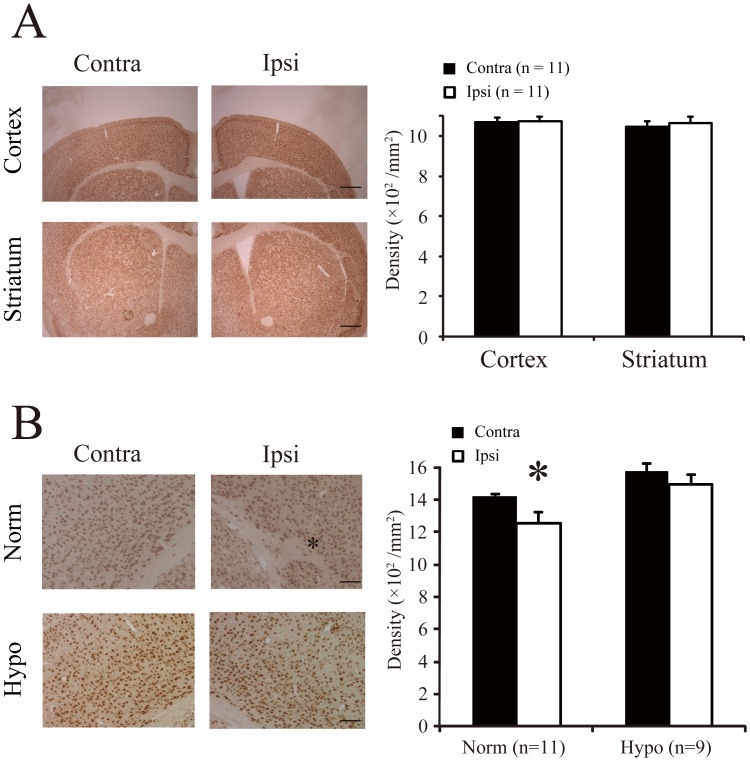
Neuronal cell density reduced in adult mouse brains after hypoxic ischemia injury. *A*: Immunohistochemistry shows NeuN-positive cells in the cortex (*Top*) and striatum (*Bottom*) of mice subjected to hypoxic ischemia injury, followed by normothermia. Scale, 500 µm. Average density of NeuN cells (n = 11 mouse brains, mean±SE) was assessed in the contralateral (black) and ischemic ipsilateral (white) hemispheres. *B*: High magnification images of the cortices of mice in normothermia and hypothermia groups. Scale, 200 µm. *P<0.05 (Wilcoxon test). Other notations are defined in (*A*).

After the behavioral tests, we performed histological analyses. We found that, in the normothermia group, the brain volume of the ischemic hemisphere was smaller than in the contralateral hemisphere. However, the densities of neurons (Fig. 3A) were not significantly different between the ischemic and contralateral hemispheres in the cortex (ipsilateral, 1076±21 cells/mm^2^; contralateral, 1070±22 cells/mm^2^; Wilcoxon test, *P* = 0.79) or the striatum (ipsilateral: 1062±32 cells/mm^2^; contralateral: 1047±28 cells/mm^2^; Wilcoxon test, *P* = 0.53). Nevertheless, at the site that the lesion was most severe, white matter fibers encroached considerably on the cortical deep layers (Fig. 3B). We found that, at those sites, the number of NeuN-positive cells was significantly lower in the ischemic cortex than in the contralateral cortex (ipsilateral: 1253±66 cells/mm^2^; contralateral: 1421±15 cells/mm^2^: Wilcoxon test, *P*<0.05). On the other hand, the hypothermia group did not show any significant differences between hemispheres, including the numbers of NeuN-positive cells at the most severe lesion sites (ipsilateral: 1493±60 cells/mm^2^; contralateral: 1579±42 cells/mm^2^; Wilcoxon test, *P*<0.06).

**Figure 4:**
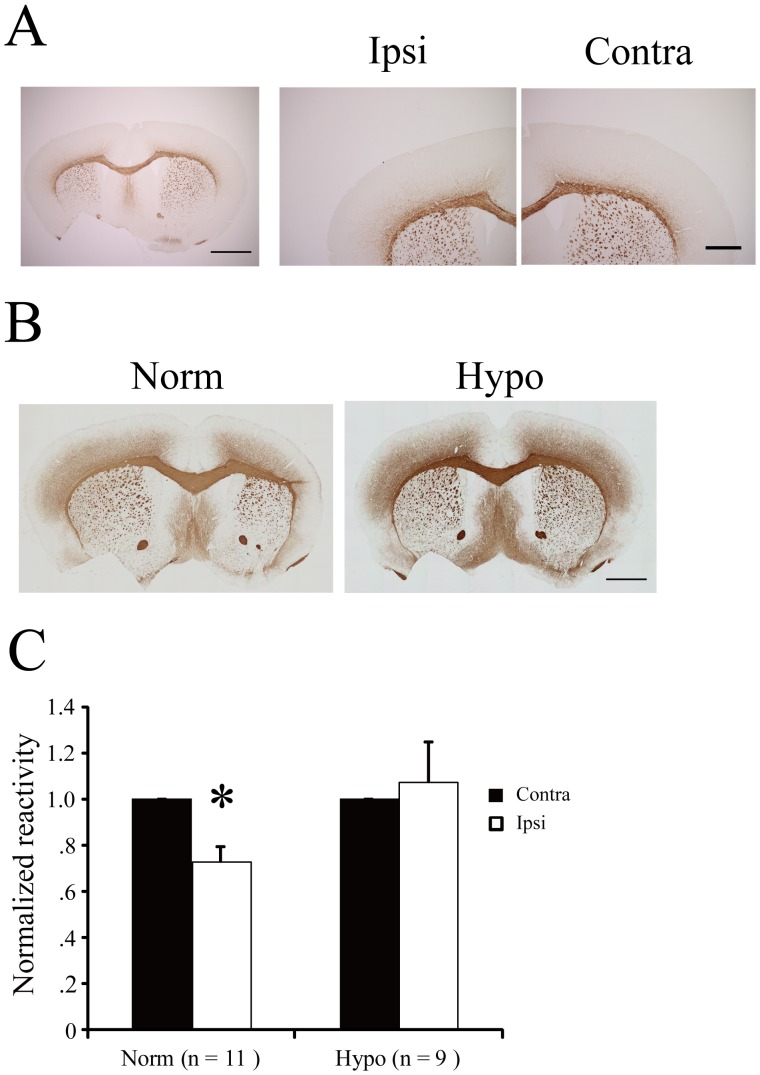
Disruption in myelination over time caused by early hypoxic ischemia injury in mouse brain. *A*: Immunohisctochemical staining of myelin binding protein (MBP) in the normothermia group at P12. Scale, 500 µm. High magnification images of the contralateral (*right*) and ipsilateral (*left*) hemisphere. Scale, 200 µm. *B*: MBP staining in the normothermia and hypothermia groups at 5 weeks. Scale, 500 µm. *C*: Normalized MBP immunoreactivity compared in normothermia (n = 11) and hypothermia (n = 9) groups. *P<0.05 (Wilcoxon test).

A previous study demonstrated that the major pathological characteristic of HI in the immature stages of embryonic development was a chronic disturbance in myelination. They suggested that oligodendrocyte progenitors were the primary targets of ischemic injury in human periventricular leukomalacia [4]. Therefore, we also investigated MBP expression with immunohistochemistry. At P12, mice had a low abundance of myelinated fibers in the cortex (Fig. 4A). In adult mice (≥5 weeks old), we found no drastic changes in the white matter. Therefore, we mainly focused on mice at 5 weeks of age to examine changes in the myelinated fibers of the cortex. Figure 4B shows that, in the normothermia group, MBP reactivity was clearly reduced in the ischemic ipsilateral cortex compared to the contralateral cortex. The MBP reactivity was quantified by calculating the ratio (ipsilateral/contralateral) of signal intensities in each animal. The normothermia group showed a significant reduction in MBP immuoreactivity (27.4% lower) in the ischemic hemisphere compared to the contralateral hemisphere (Fig. 4C; Wilcoxon test, *P*<0.01). In contrast, the hypothermia group showed no significant difference between the MBP signal intensities in the two hemispheres (Fig. 4C; Wilcoxon test, *P* = 0.80).

**Figure 5:**
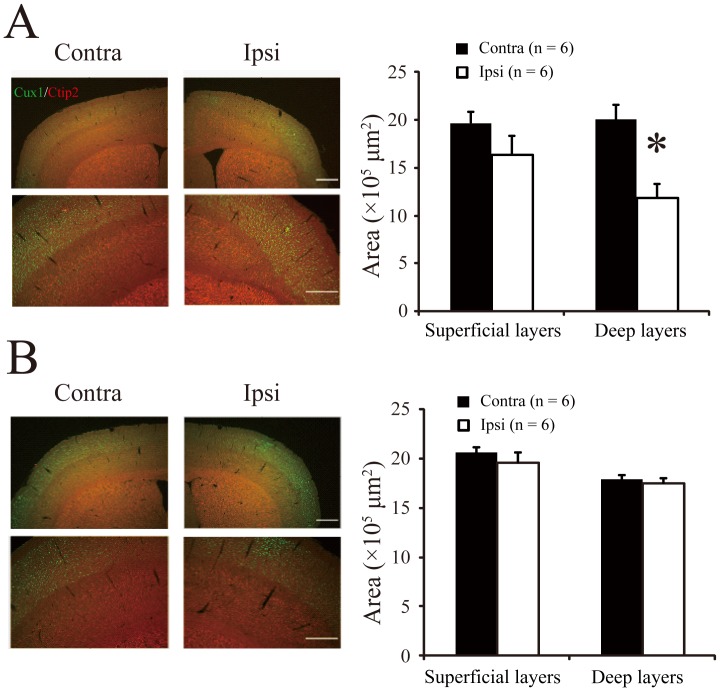
Disruptions in laminar structure of mouse brain cortex after hypoxic ischemia injury. *A*: (*left*) Histological tissue sections show the laminar structure of the cortex in the normothermia group. Scale bar, 500 µm. The designated areas are enlarged in the bottom panels for clarity. Scale bar, 200 µm; *(right*) average areas of superficial and deep cortical layers measured on mouse brain sections (n = 6, mean±SE). *P<0.05 (Wilcoxon test). *B:* Laminar structure in the hypothermia group (n = 6, mean±SE). Other notations defined in (*A*).

Histologically, laminar disruption was observed within 7 days after normothermia treatment (data not shown). Therefore, after the behavioral tests at 4 weeks, we performed double immunofluorescence staining for Ctip2 and Cux1 to visualize the deep and upper cortical layers, respectively. The normothermia group showed a distinct loss of volume concomitant with structural changes in the deep (V/VI) cortical layers. The deep layer thicknesses were 1.13×10^6^ µm^2^ on the ipsilateral side and 1.90×10^6^ µm^2^ on the contralateral side (Fig. 5A; Wilcoxon test, *P*<0.05). In contrast, in the hypothermia group, these developmental disturbances were not observed; the laminar structures were similar in both hemispheres (Fig. 5B, deep layer thicknesses, ipsilateral: 1.75×10^6^ µm^2^; contralateral: 1.80×10^6^ µm^2^; Wilcoxon test, *P* = 0.35).

**Figure 6:**
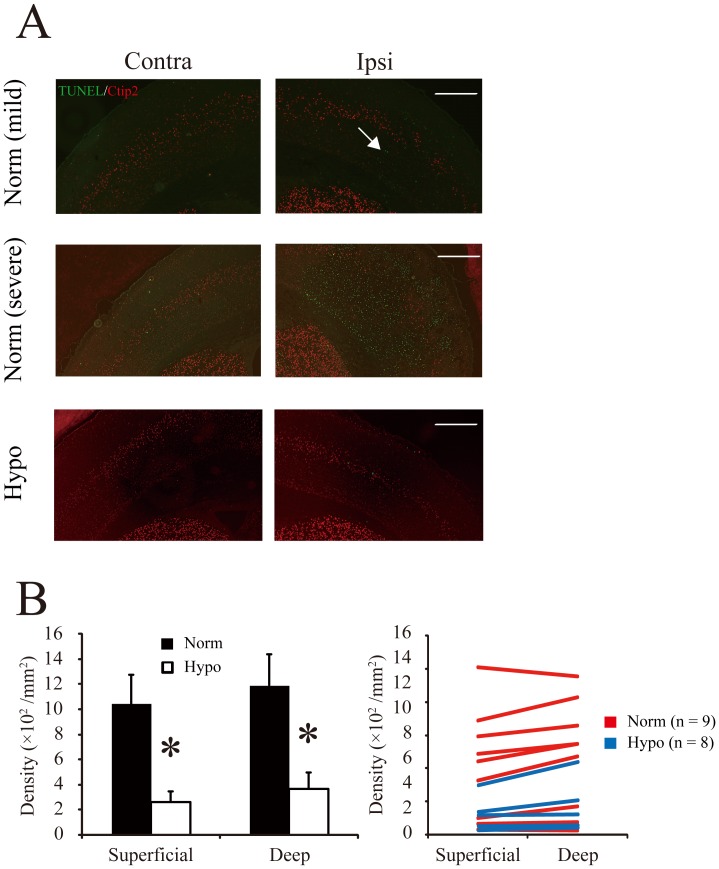
Apoptosis (TUNEL staining) in the cortex 24 h after hypoxic ischemia injury. *A*: Tissue sections are shown from brain hemispheres subjected to mild ischemic injury (*top*) or severe ischemic injury in mice from the normothermia (*middle*) and hypothermia (*bottom*) groups. *Arrow* indicates TUNEL-positive cells. Scale, 500 µm. *B*: *(left*) Average number of TUNEL-positive cells (mean±SE) in the superficial and deep cortical layers in the normothermia (n = 9) and hypothermia (n = 8) groups. *P<0.05 (Wilcoxon test). *(Right*) Distribution of the number of TUNEL-positive cells in the superficial layers and deep cortical layers.

To assess cell death, we labeled cells with TUNEL staining to detect pyknotic nuclei immediately after the normothermia/hypothermia therapy. In the normothermia group, mild ischemia induced apoptotic cells predominantly in the deep cortical layers, but severe ischemia induced extensive destruction. The ischemic cortex had a smaller number of TUNEL-positive cells in the hypothermia group than in the normothermia group (Fig. 6A). To compare the numbers of TUNEL-positive cells between the superficial and deep cortical layers, we performed double immunofluorescence staining with anti-Ctip2 and TUNEL. Although the distribution of TUNEL-positive cells was dependent largely on the extent of the injury, cell death was predominantly detected in the deep cortical layers (Fig. 6B). Compared to normothermia, hypothermia showed significantly reduced TUNEL-positive cell densities in both the upper cortical layer (normothermia: 1040±235/mm^2^; hypothermia: 263±87/mm^2^; Wilcoxon test, *P*<0.05) and the deep cortical layer (normothermia: 1188±248/mm^2^; hypothermia: 367±130/mm^2^; Wilcoxon test, *P*<0.05).

## Discussion

In this study, we used P3 mice rather than the P7 mice typically used in previous studies [23], because we aimed to examine hypoxia in immature brain, equivalent to that of an early, third-trimester human fetus [24]. We considered this P2–3 model with ischemia appropriate for investigating conditions that can lead to CP. The results showed that neonatal HI followed by normothermia induced: 1) laminar abnormality in the cortex, 2) focal necrosis in the deep cortical layers, and 3) reduced myelination in the cortex at an adult age. Furthermore, our results suggested that hypothermia therapy might improve these adverse outcomes by attenuating neural cell death.

### Cortical Laminar Necrosis

In the case of sample with severe brain injury, neural death was evident in both the superficial and deep layers immediately after normothermia therapy. However, mild ischemic brain injury predominantly induced neural death in the deeper layers. These results suggested that there may be region-specific susceptibility to ischemia injury. This may, in part, be attributed to the fact that blood vessels are poorly developed in the immature brain at P2–3 [25], [26]; thus, the deep region may be particularly susceptible to hypoxia due to a limited blood supply. In addition, previous *in vitro* cell culture studies with embryonic cells demonstrated that a progressive increase in the susceptibility to ischemia could evolve in parallel with the expression of NMDA receptor subunits [27], [28]. Since the deeper layers develop morphologically and functionally before the relatively younger outer layers (“inside-out” development) [29], [30], neuronal immaturity in the outer cortical layers may have contributed to protection from small infarcts.

Selective vulnerability to ischemic injury may result in cortical laminar disturbances in the adult. Our results were consistent with prior investigations that demonstrated that HI in the neonatal immature brain was associated with selective injury in the subplate neurons, but most of the cortical neurons remained intact [31], [32]. In contrast, Stadlin *et al.* demonstrated that TUNEL-positive cells were distributed in the upper cortical layers 3 days after HI [33]. The discrepancy may be related to a difference in the amount of time following the injury. Indeed, we observed a reduction in Ctip2-positive neurons at 24 h after HI; this result suggested that cortical neurons in the deep layer had died. A potential explanation for the discrepancy could be that the infarct area expanded from the deep layers to superficial layers over time. We also detected Cux1-positive cells in the deep layers. Thus, it is possible that neurons that should have formed part of the superficial layers could have migrated to the deep layers, because at P2–3, the cortical layers were not fully constructed [29]. On the other hand, the focal core necrosis remained, consistent with the previous report that HI induced irreversible ischemic brain damage that resulted in the death of radial column cells [34].

### Myelination Disorder

Previous clinical and experimental data have shown that immature oligodendrocytes were highly susceptible to HI injury [35], [36]. The white matter of the P2 rodent contains predominantly pre-oligodendrocytes; thus, early rodent white matter resembles human periventricular white matter during the high-risk period for periventricular white matter injury [37], when significant myelination is induced. Our data suggested that hypothermia attenuated the HI-induced reduction of myelin immunoreactivity in the neocortex. To our knowledge, this was the first report to demonstrate that hypothermia was associated with improved myelination in adult mice after HI. The changes we observed in MBP immunoreactivity were most likely linked to oligodendrocyte survival, because the density of NeuN-positive cells did not change after ischemia. However, we could not rule out the possibility that both neurons and axons could have been injured or destroyed in the cortex during postnatal HI. Further work is necessary to investigate the extent to which neuronal and/or axonal loss was due to HI in the premature brain.

### Hypothermia

This was the first study to demonstrate that hypothermia in very immature mouse brain could attenuate HI-induced laminar disruption in the deep cortical layers. Brain development in the 2–3 day rodent pup has been likened to that in a very premature infant (less than 32 weeks gestation) [38]. However, it has also been shown that hypothermia is a risky treatment for preterm babies [39].

Although the precise mechanisms underlying the effects of hypothermia have not been elucidated, low brain temperature has been shown to be neuroprotective against glutamatergic excitotoxicity [20], and it counteracted suppression of synaptic transmission [40]. The present study showed that hypothermia-induced protection of cortical neurons was mediated by prevention of apoptosis, consistent with previous studies [19], [41]. In addition, a recent study demonstrated that hypothermia in combination with a *casp2* gene deletion enhanced neonatal brain protection against HI [42]. Based on our preliminary study, a reduced brain temperature was associated with reductions in metabolism, cerebral blood flow, and glutamate release [43]. Therefore, it is reasonable to hypothesize that hypothermia treatment may also be effective for treating newborn infants exposed to HI. However, clinical trials that tested hypothermia for treating preterm infants with brain injuries have reported varying results [44].

With the exception of infectious complications, this is partly because the direct molecular mechanisms underlying hypothermic neuroprotection via thermo-sensitive channels remain unclear. A potential mechanism was proposed that involved inactivation of ion channels known as the transient receptor potential cation channel subfamily V (TRPV3/TRPV4). Inactivation of these channels by moderate changes in temperature may be partly responsible for the neuroprotective effect of hypothermia [45]. Furthermore, TRPV1 agonists have been demonstrated to induce hypothermia in rats [46], [47]. Further research into the role of TRPV channels is needed to understand whether TRPV modulation can be effectively applied to clinical conditions.

In conclusion, our results from preclinical tests suggested that hypothermia may be a useful method for treating very premature infants (born before 36 weeks gestation) for HI-induced brain damage.
